# Role of long non‐coding RNAs in cholangiocarcinoma: A systematic review and meta‐analysis

**DOI:** 10.1002/cnr2.2029

**Published:** 2024-03-22

**Authors:** Parnian Shobeiri, Razman Arabzadeh Bahri, Mohamad Mehdi Khadembashiri, Mohamad Amin Khadembashiri, Saba Maleki, Mohammad Eslami, Marzie Khalili Dehkordi, Amir Hossein Behnoush, Nima Rezaei

**Affiliations:** ^1^ School of medicine Tehran University of Medical Sciences Tehran Iran; ^2^ Network of Immunity in Infection, Malignancy and Autoimmunity (NIIMA), Universal Scientific Education and Research Network (USERN), Tehran Iran; ^3^ Research Center for Immunodeficiencies, Pediatrics Center of Excellence, Children's Medical Center Tehran University of Medical Sciences Tehran Iran; ^4^ Urology Research Center Tehran University of Medical Sciences Tehran Iran; ^5^ Neuromusculoskeletal Research Center Iran University of Medical Sciences Tehran Iran; ^6^ Student Scientific Research Center (SSRC) Tehran University of Medical Sciences Tehran Iran; ^7^ School of Medicine Guilan University of Medical Sciences Rasht Iran; ^8^ Department of Immunology, School of Medicine Tehran University of Medical Sciences Tehran Iran

**Keywords:** Cholangiocarcinoma, diagnosis, long non‐coding RNA, meta‐analysis, systematic review

## Abstract

**Background:**

Cholangiocarcinoma (CCA), as a rare malignancy of the biliary tree, has a poor prognosis most of the time. CCA is highly epigenetically regulated and several long non‐coding RNAs (lncRNA) have been investigated to have a diagnostic and prognostic role in CCA. The current study aimed to assess the studies finding relevant lncRNAs in CCA systematically.

**Methods:**

International databases, including PubMed, Cochrane Library, and Embase, were comprehensively searched in order to identify studies investigating any lncRNA in CCA. After screening by title/abstract and full‐text, necessary data were extracted. Random‐effect meta‐analysis was performed for pooling the areas under the curve (AUCs), specificity, and sensitivity of lncRNAs for the diagnosis of CCA.

**Results:**

A total of 33 studies were chosen to be included in the final analysis, comprised of 2677 patients. Meta‐analysis of AUCs for evaluation of CCA resulted in pooled AUC of 0.79 (95% CI: 0.75–0.82; *I* ^2^ = 69.11, *p* < .01). Additionally, overall sensitivity of 0.80 (95% CI 0.75–0.84) and specificity of 0.77 (95% CI: 0.68–0.84) were observed. Measurement of lncRANs in the assessment of CCA also improved overall survival significantly (effect size 1.61, 95% CI: 1.39–1.82). A similar result was found for progression‐free survival (effect size 1.57, 95% CI: 1.20–1.93).

**Conclusion:**

Based on our findings, lncRNAs showed promising results as biomarkers in the diagnosis of CCA since they had acceptable sensitivity and specificity, in addition to the fact that improved survival in this poor prognosis cancer. Further studies might be needed to address this issue and find the best clinically useful lncRNA.

## 1 INTRODUCTION

Cholangiocarcinoma (CCA) is a large variety of malignancies that can arise at any point along the biliary tree, from the Hering canals to the common bile duct.^1^ CCA is a rare malignancy, but its incidence and mortality rates have been rising in the past few decades. With a global incidence rate of 0.3–6/100000 people per year and 1–6/100000 mortality rate annually, certain regions have an incidence rate of over 6/100000 inhabitants per year.^2^ Despite considerable progress in disease awareness, knowledge, diagnosis, and treatments, no enhancement has been observed in prognosis over the past 10 years since 5‐year survival rates range from 7% to 20%, in addition to disappointing tumor recurrence rates after resection.^3^ The diagnosis of diseases in the early stages is difficult since no specific clinical presentation exists. A majority of patients do not show significant clinical manifestations until the progression of the disease to late intermediate or advanced stages at which point therapeutic choices are highly restricted, and the prognosis is poor.^4^

Epigenetic regulations are highly involved in CCA. Histone modification, non‐coding ribonucleic acids (RNAs), and deoxyribonucleic acid (DNA) methylation are standard epigenetic processes in gene regulation. However, not all of these processes have been thoroughly investigated in studies on human CCA.^5^ The crucial step in improving the prognosis of CCA patients would be investigating early diagnostic indicators with high sensitivity and developing specific therapeutic medications. To find novel biomarkers and therapeutic targets for CCA, it is necessary to assess the critical molecular pathways needed for the initiation and progression of the disease.^6^

About 75% of the human genome is usually transcribed into RNA. This is while only 3% is transcribed into messenger RNAs (mRNAs) that code for proteins. Non‐coding RNAs (ncRNAs) can be classified into different types with different lengths, shapes, and locations. Long ncRNA (lncRNA), PIWI‐interacting RNA (piRNA), microRNA (miRNA), and circular RNA (circRNA) are the four types with specialized roles in several diseases and malignancies.^7^, ^8^, ^9^, ^10^ Across a broad spectrum of cancers, carcinogenic or tumor‐suppressing lncRNAs' aberrant expressions have been reported. LncRNA‐associated epigenetic regulation has also been discovered in the molecular processes of CCA development.^6^ In addition, as epigenetic process modulators, lncRNAs can regulate gene expression in several ways. These characteristics indicate the importance of lncRNA applications in cancer diagnosis, prognosis, and treatment.^11^

Although different results and different accuracies were reported by studies in the literature, to date, no systematic review has pooled the results obtained by individual studies assessing the diagnostic and prognostic ability of lncRNAs in CCA, and hence, no definitive conclusion could be made by these studies alone. The current study synthesizes the ever‐increasing studies investigating the correlation between the expression of lncRNA with CCA and their diagnostic and prognostic values in clinical settings via systematic review and meta‐analysis.

## 2 METHODS AND MATERIALS

### 2.1 Search strategy

This study was performed according to the PRISMA guidelines (Preferred Reporting System for Systematic Reviews and Meta‐analyses).^12^ We performed a comprehensive search in order to identify all of the published articles regarding the long non‐coding RNAs and CCA in international bibliometric databases, including PubMed, Cochrane Library, and Embase, from inception until October 2022. No limitations on the original language of the studies were imposed. A second database search was conducted 1 week before the submission of the present article in order to identify newly published papers regarding this topic.

### 2.2 Eligibility criteria

The inclusion criteria for study selection were: (a) CCA patients for which CCA was histopathologically confirmed, (b) reported lncRNA's expression levels categorized into high and low and the reported correlation between expression of lncRNA and clinical and pathological features; (c) the reported association between lncRNA expression and survival outcome, hazard ratios (HR), 95% confidence intervals (CI), *p*‐values, and Kaplan–Meier curves, and (d) the reported expression of lncRNAs obtained from the tissue and/or serum; and (e) data on sensitivity, specificity, and sample sizes. The exclusion criteria for study selection were: (a) non‐human and animal studies; (b) letters to editors, case report studies, commentary studies, congress abstracts, or review articles; (c) articles not related to lncRNA and CCA; (d) inadequate available data for conducting a meta‐analysis based on the items of excel sheet for data extraction; (e) HRs calculated using several (other than one) lncRNAs, and (f) studies investigating lncRNAs genetic polymorphisms.

### 2.3 Data extraction and quality assessments

Data extraction was conducted by two authors, independently, based on a predefined excel sheet, including the first author's name, publication year, type of lncRNA, study participants, age and gender of patients, tumor size, grade and stage of cancer, detection method, sensitivity, specificity and area under the curve (AUC) of diagnostic studies, HRs, 95% CIs, and *p*‐values for survival analysis. In case of the absence of patient data, we used the Kaplan–Meier curves method based on the work by Tierney et al.^13^ or requested from their primary authors. Two authors independently conducted using the quality assessment of diagnostic accuracy studies tool version 2 (QUADAS‐2) and the quality in prognostic studies tool (QUIPS) for diagnostic accuracy studies and prognostic studies, respectively.

### 2.4 Statistical analysis

The chi‐square test and *I* ^2^ statistic were used to assess heterogeneity among articles. An *I* ^2^ higher than 50% was considered a significant heterogeneity. A random‐effect model was used when high heterogeneity was observed in studies. Otherwise, a fixed‐effect model was utilized to evaluate the relationship between different lncRNA expressions and survival outcomes.

Three different effect sizes were used for meta‐analyses. For the diagnostic meta‐analysis, we used sensitivity, specificity, and AUC. For clinicopathological features, we used odds ratios (ORs) and their 95% CIs, and we performed a meta‐analysis using HRs and associated 95% CIs for prognostic studies. The pooled AUC was determined by meta‐analysis to determine the diagnostic capability of lncRNAs in CCA. Moreover, pooled sensitivities, specificities, positive likelihood ratios, and negative likelihood ratios were calculated by meta‐analysis of individual studies. The AUC of summary receiver operating characteristic (SROC) for lncRNAs in CCA was also calculated. Finally, the effect sizes for overall survival (OS) and progression‐free survival (PFS) were pooled and the overall OS and PFS were calculated.

Random‐effect meta‐analysis was used for performing meta‐analyses and Higgins' *I*‐square test based on Cochrane's *Q* was used for the assessment of heterogeneity. The thresholds used for heterogeneity (*I* ^2^) were ≤ 25%, 26–75%, and ≥ 75% for low, moderate, and high heterogeneity, respectively.

For assessment of each study's effects on the pooled estimate, sensitivity analysis by the leave‐one‐out method was performed. For the evaluation of possible publication bias, a visual assessment of funnel plots and Deek's funnel plots was performed. All statistical analyses and graphical designs were conducted using R version 4.2.1 (R core team, 2022) with the meta package, and Stata software (Stata Corp. version 17.0). A *p*‐value of .05 or below was considered significant.

## 3 RESULTS

### 3.1 Identification of studies

**FIGURE 1:**
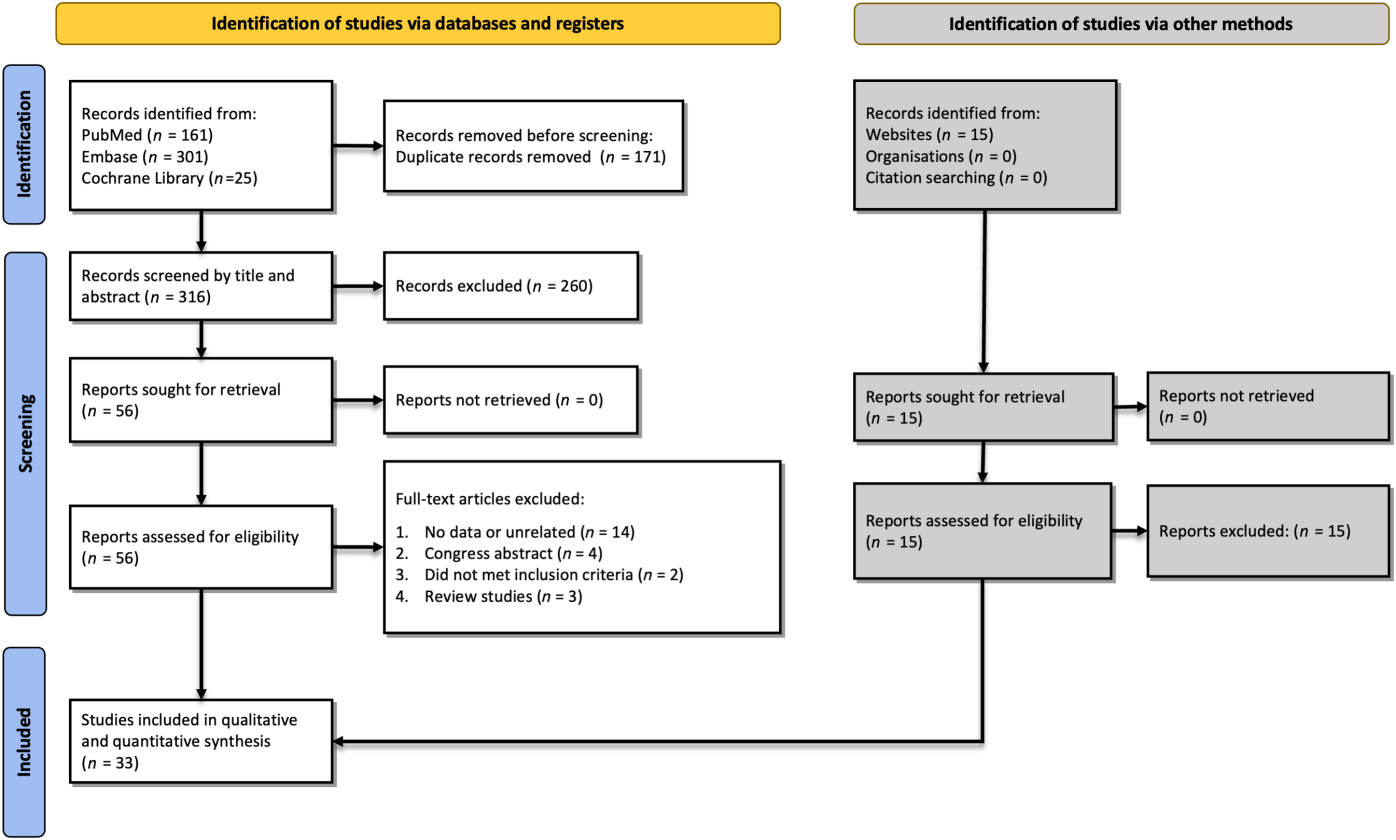
Preferred Reporting System for Systematic Reviews and Meta‐analyses flowchart of the literature search and selection of the articles.

A total of 316 articles were obtained from database searches. After duplicate removal, 258 studies remained for screening based on titles/abstracts. We excluded (*n* = 202) studies based on our eligibility criteria. Therefore, (*n* = 56) full‐text articles were reviewed, and (*n* = 33) studies were included finally.^14^, ^15^, ^16^, ^17^, ^18^, ^19^, ^20^, ^21^, ^22^, ^23^, ^24^, ^25^, ^26^, ^27^, ^28^, ^29^, ^30^, ^31^, ^32^, ^33^, ^34^, ^35^, ^36^, ^37^, ^38^, ^39^, ^40^, ^41^, ^42^, ^43^, ^44^, ^45^, ^46^ Figure 1 represents the PRISMA flowchart and the reasons for exclusion.

### 3.2 Study characteristics

The characteristics of the included studies are shown in Tables 1 and 2. They were published between 2017 and 2022. A total of 2677 patients were evaluated in 31 of the included studies, and two studies had unknown sample sizes. All of the included studies were carried out in China. Most of the studies reported the age of the population with a cutoff of 60 years old, as shown in Tables 1 and 2. The diagnostic accuracy of measurement of lncRNAs for CCA was evaluated in 10 studies.^17^, ^19^, ^20^, ^21^, ^23^, ^27^, ^30^, ^31^, ^33^, ^34^ Supplementary Table 2 presents the quality assessment results of the included studies based on QUADAS‐2 or QUIPS tools.

**TABLE 1:** Summary of lncRNAs used as diagnostic biomarkers of cholangiocarcinoma.

Study	Location	lncRNA	Function	Case	Control	Age	Sex (male)	Expression	AUC	Sensitivity	Specificity
Canghai Guan (2020)^19^	China	SNHG20	Oncogene	25	24	Case: 36% (<60), 64% (≥ 60) Control: 46% (<60), 54% (≥ 60)	Case: 36% Control: 50%	Upregulation	0.7484 (0.6515–0.8454)	73.4	75.5
Bo‐Wei Han (2018)^20^	China	AC005550.3	Oncogene	36	‐	NR	NR	Upregulation	0.7695 (0.6015–0.9375)	93.75	81.25
Zengtao Hu (2022)^21^	China	FOXD2‐AS1	Oncogene	28	29	Case: 39% (<60), 61% (≥60) Control: 45% (<60), 55% (≥60)	Case: 57% Control: 48%	Upregulation	0.7406 (0.6447‐ 0.8365)	82	60
Xianxiu Ge (2017)^17^	China	ENST00000588480.1	Oncogene	35	‐	NR	NR	Upregulation	0.68 (0.565–0.796)	62.9	73.2
Jinglin Li (2020)^27^	China	HOXD‐AS1	Oncogene	29	27	Case: 31% (<60), 69% (≥60) Control: 37% (<60), 63% (≥60)	Case: 48% Control: 41%	Upregulation	0.786 (0.668–0.904)	80	65
Chunying Luo (2021)^31^	China	LINC02560	Oncogene	309	310	Total: 43% (≤ 65), 57% (>65)	Total: 53%	Upregulation	0.763 (0.723–0.803)	‐	‐
Jinglin Li (2019)^26^	China	MEG3	Oncogene	30	30	Case: 30% (<60), 70% (≥60) Control: 33% (<60), 67% (≥60)	Case: 47% Control: 40%	Upregulation	0.791 (0.674–0.908)	75.80	77.80
Zhanqiang Liang (2019)^30^	China	NEF	Tumor suppressor	56	42	Case: 52.1 ± 6.1 Control: 51.4 ± 5.8	Case: 75% Control: 71%	Downregulation	0.8642 (0.7932–0.9351)	‐	‐
Dongsheng Sun (2022)^34^	China	PSMA3‐AS1	Oncogene	38	28	Case: 21% (<60), 79% (≥60) Control: 39% (<60), 61% (≥60)	Case: 39% Control: 50%	Upregulation	0.793 (0.668–0.919)	‐	‐
Jian Shi (2018)^33^	China	CPS1IT1	Oncogene	20	20	NR	NR	Upregulation	0.66 (0.491–0.828)	‐	‐
Jian Shi (2018)^33^	China	PCAT1	Oncogene	20	20	NR	NR	Upregulation	0.802 (0.651–0.954)	‐	‐
Jian Shi (2018)^33^	China	MALAT1	Oncogene	20	20	NR	NR	Upregulation	0.795 (0.644–0.947)	‐	‐

Abbreviations: AUC, area under curve; NR, not reported.

**TABLE 2:** Summary of lncRNAs used as prognostic biomarkers of cholangiocarcinoma.

Study	Location	LncRNA	Sample size	Age	Sex (male)	Expression	Survival analysis	HR	*p*‐Value
Canghai Guan (2020)^19^	China	SNHG20	49	41% (<60) 59% (≥60)	43%	Upregulation	OS	2.315 (1.304–4.312)	.012
Lining Huang (2020)^22^	China	Linc000473	60	32% (<60) 68% (≥60)	43%	Upregulation	OS	2.365 (1.34–4.173)	.013
Yulei Gu (2019)^18^	China	NNT‐AS1	89	48% (≤55) 52% (>55)	55%	Upregulation	OS	2.108 (1.561–2.594)	.025
Jian‐Guo Bai (2018)^14^	China	CCAT2	106	47% (<60) 53% (≥60)	74%	Upregulation	OS	3.184 (1.882–5.385)	<.001
							PFS	2.926 (1.771–4.834)	<.001
Jianjun Gao (2020)^15^	China	Linc00261	50	52% (<60) 48% (≥60)	34%	Upregulation	OS	2.300 (1.189–4.451)	.013
Zengtao Hu (2022)^21^	China	FOXD2‐AS1	57	42% (<60) 58% (≥60)	53%	Upregulation	OS	2.241 (1.337–3.879)	.01
Xiu‐Liang Xia (2018)^37^	China	CRNDE	118	49% (<60) 51% (≥60)	68%	Upregulation	OS	1.365 (1.221–1.525)	<.001
							PFS	1.366 (1.224–1.525)	<.001
Xiaozai Xie (2021)^38^	China	five lncRNA (HULC; AL359715.5; AC006504.8; AC090114.2; AP00943.4)	36	25% (<60) 75% (≥60)	56%	AL359715.5: Downregulation The others: Upregulation	OS	6.760 (1.572–29.068)	.008
Yi Xu (2021)^39^	China	Circ‐LAMP1	216	31% (<48) 69% (≥48)	59%	Upregulation	OS	2.300 (1.588–3.332)	<.001
							DFS	2.257 (1.609–3.166)	<.001
Yi Xu (2017)^40^	China	PANDAR	67	61% (<60) 39% (≥60)	44%	Upregulation	OS	2.228 (1.269–3.913)	.005
Yi Xu (2018)^41^	China	SPRY4‐IT1	70	58% (<60) 42% (≥60)	45%	Upregulation	OS	1.876 (1.073–3.280)	.027
							PFS	1.970 (1.122–3.461)	.018
Yi Xu (2017)^42^	China	UCA1	68	59% (<60) 41% (≥60)	44%	Upregulation	OS	2.410 (1.403–4.149)	.001
Bing Zeng (2017)^44^	China	TUG1	102	NR	NR	Upregulation	OS	2.44 (1.57–3.78)	<.001
							DFS	2.04 (1.37–3.04)	<.001
Bingquan Zhang (2019)^45^	China	LOXL1‐AS1	64	50% (<60) 50% (≥60)	48%	Upregulation	OS	2.293 (1.308e4.022)	.004
Dongkai Zhou (2019)^46^	China	HULC	81	NR	NR	Upregulation	OS	1.22 (1.02–1.47)	.023
Zhendong Li (2018)^29^	China	ANRIL	82	50% (<60) 50% (≥60)	57%	Upregulation	OS	1.403 (1.146–1.717)	.001
							PFS	1.346 (1.113–1.627)	.002
Jinglin Li (2020)^27^	China	HOXD‐AS1	56	34% (<60) 66% (≥60)	45%	Upregulation	OS	2.124 (1.193–3.780)	.01
Jinglin Li (2021)^25^	China	LINC00667	62	29% (<60) 71% (≥60)	44%	Upregulation	OS	2.014 (1.118–3.629)	.02
Jinglin Li (2019)^26^	China	MEG3	60	32% (<60) 68% (≥60)	43%	Upregulation	OS	2.127 (1.204–3.755)	.009
Dongsheng Sun (2021)^35^	China	PCAT1	56	30% (<60) 70% (≥60)	39%	Upregulation	OS	2.158 (1.142–4.078)	.018
Dongsheng Sun (2022)^34^	China	PSMA3‐AS1	66	29% (<60) 71% (≥60)	44%	Upregulation	OS	2.059 (1.159–3.685)	.014
Zhenglong Li (2018)^28^	China	Sox2ot	58	53% (<60) 47% (≥60)	43%	Upregulation	OS	2.936 (1.612–5.349)	.0004
Lei Kong (2019)^24^	China	UCA1	22	NR	NR	Upregulation	OS	2.77 (0.97–7.59)	.048
Xingming Jiang (2020)^23^	China	ZEB1‐AS1	54	31% (<60) 69% (≥60)	43%	Upregulation	OS	2.220 (1.202–4.101)	.011
Daguang Tian (2019)^36^	China	SNHG3	52	56% (≤60) 44% (>60)	52%	Upregulation	OS	3.25 (1.54–7.51)	.009
Wei Qin (2018)^32^	China	HOTAIR	70	NR	NR	Upregulation	OS	2.251 (1.260–4.022)	.006
Meihong Gao (2022)^16^	China	HOTAIR	‐	NR	NR	Upregulation	OS	0.97 (0.95–0.99)	.026
		CTD‐2357A8.3	‐			Upregulation	OS	0.97 (0.95–0.99)	.007 2
		GS1‐600G8.5	‐			Upregulation	OS	0.99 (0.98–1)	.012
Yi Xu (2018)^43^	China	CCAT2	60	53% (<60) 47% (≥60)	45%	Upregulation	OS	2.015(1.137–3.570)	.016

Abbreviations: DFS, disease‐free survival; HR, hazard ratio; NR, not reported; OS, overall survival; PFS, progression‐free survival.

### 3.3 Meta‐analysis

#### 3.3.1 AUC, sensitivity, and specificity

**FIGURE 2:**
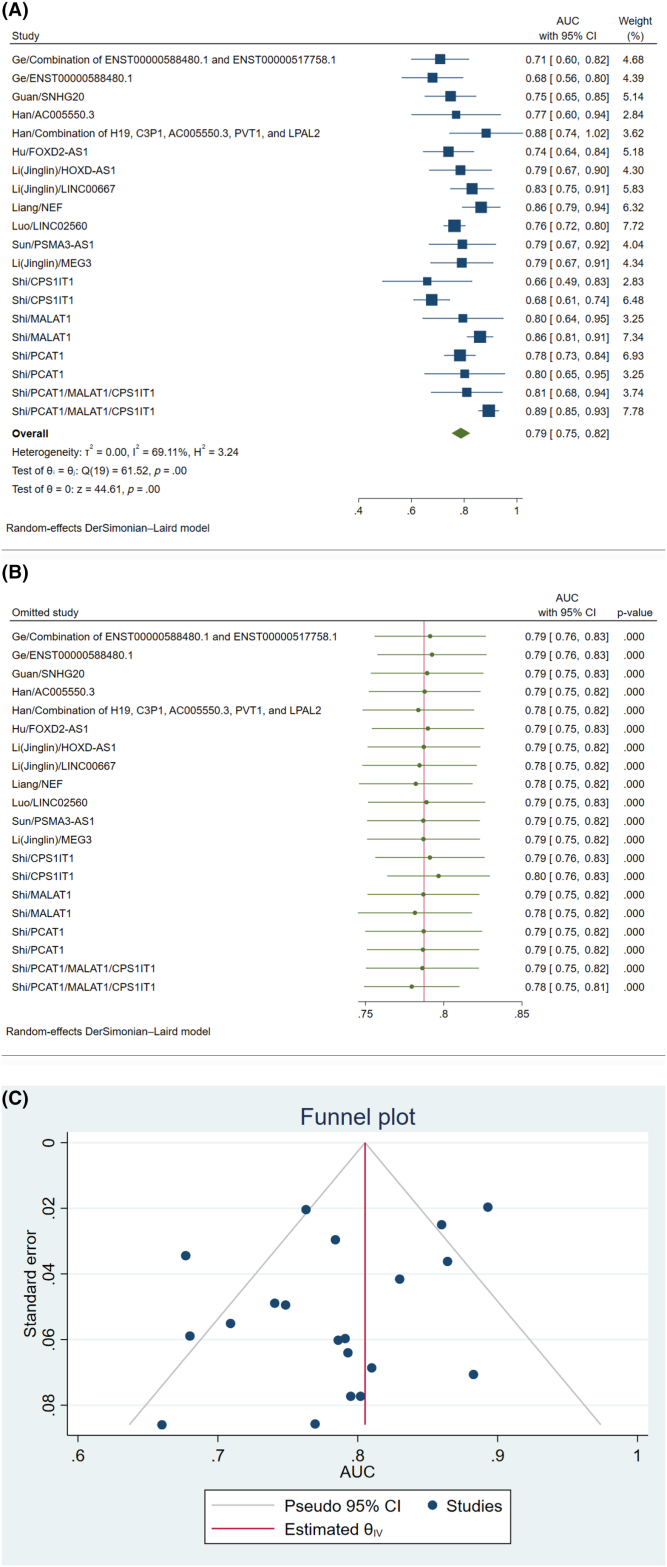
Area under the curve (AUC) of measurement of lncRNAs for assessment of cholangiocarcinoma; (A): forest plot; (B): leave‐one‐out chart; (C): funnel plot.

AUC was assessed for several lncRNAs for diagnosis of CCA. Among the studies, Shi et al. found that a combination of PCAT1, MALAT1, and CPS1IT1 had the highest AUC (0.89).^33^ This was followed by the combination of H19, C3P1, AC005550.3, PVT1, and LPAL2 in the report by Han et al. who found a diagnostic AUC of 0.88 for this combination.^20^ Based on the meta‐analysis performed, the pooled AUC of measuring lncRNAs for evaluation of CCA was 0.79 (95% CI: 0.75–0.82; *I* ^2^ = 69.11%, *p* < .01) (Figure 2A). In addition, we conducted a leave‐one‐out analysis of AUC to identify the possible source of heterogeneity in the meta‐analysis of lncRNA measurements for the evaluation of CCA (Figure 2B). None of the studies could affect the overall result significantly. There was no possible source of small study effects based on visual inspection of the funnel plot of AUC (Figure 2C).

**FIGURE 3:**
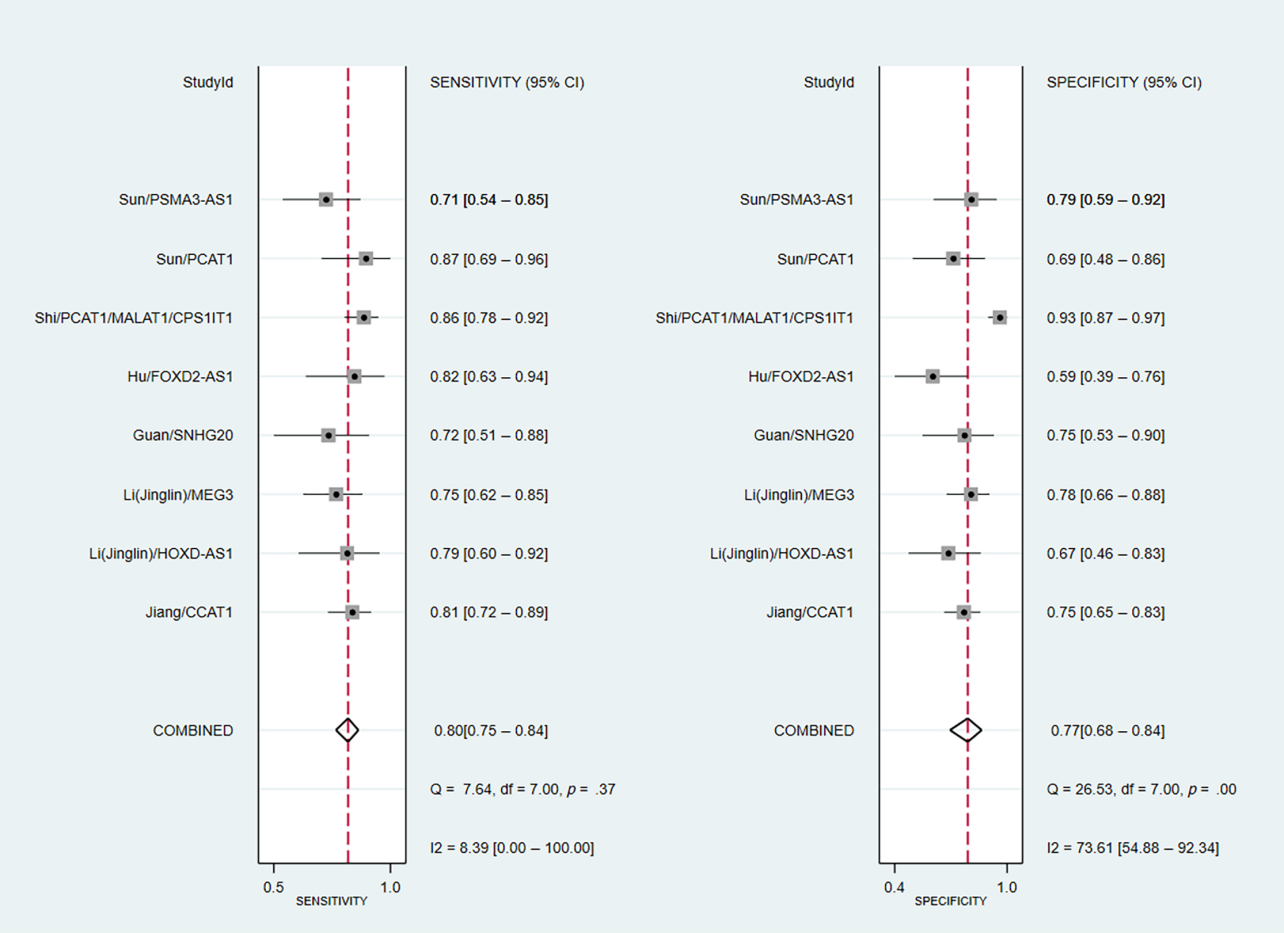
Forest plot of sensitivity and specificity of lncRNA measurements for assessment of cholangiocarcinoma.

**FIGURE 4:**
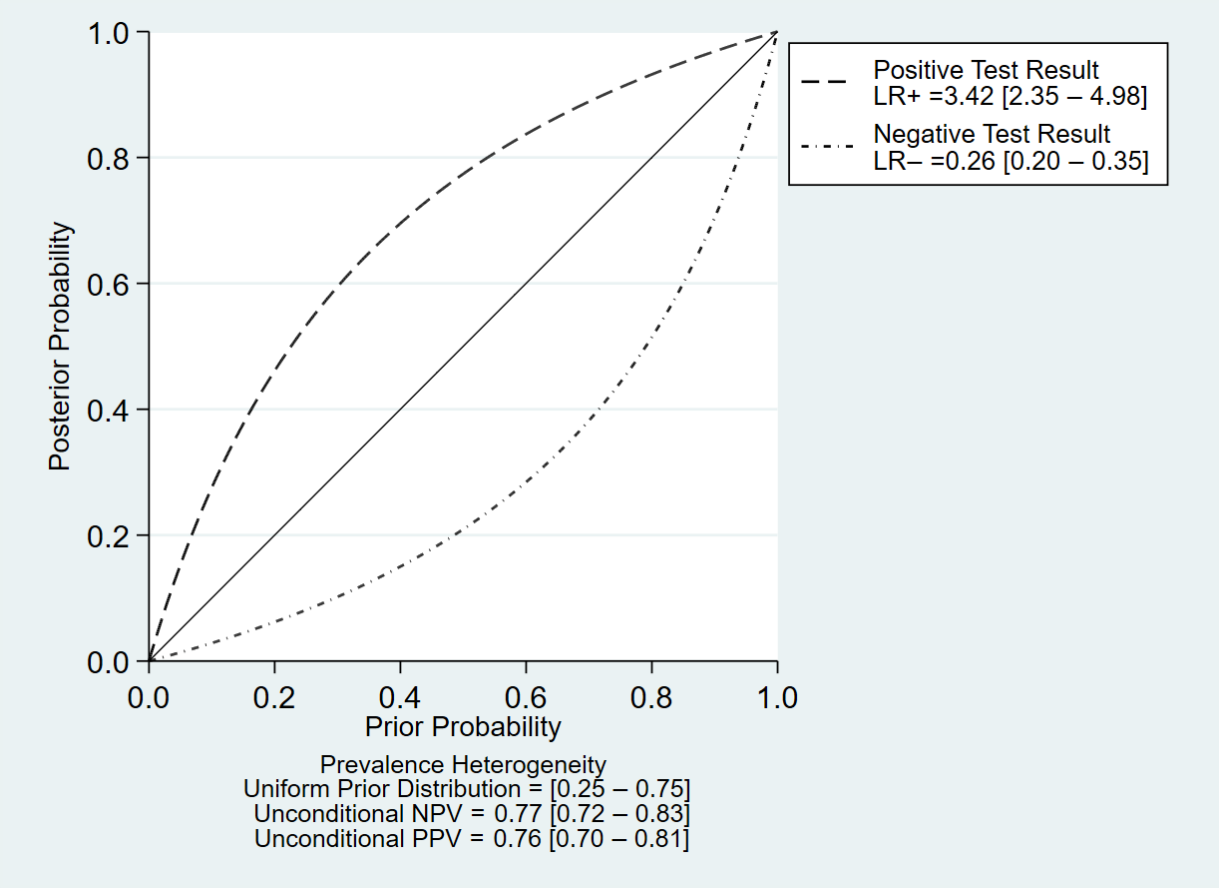
Positive and negative likelihood ratios of lncRNA measurements for assessment of cholangiocarcinoma.

**FIGURE 5:**
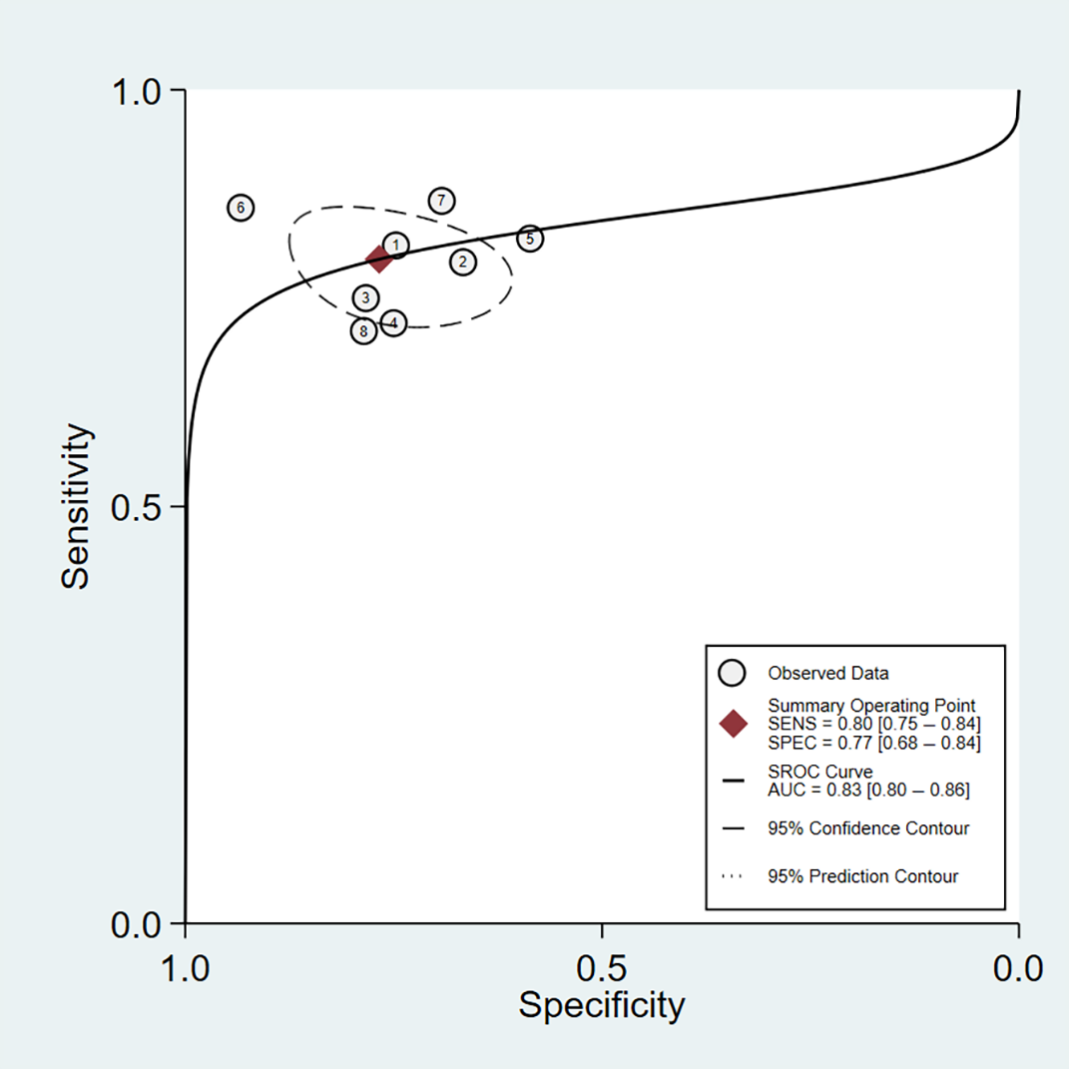
Summary receiver operative curve for measurement of lncRNAs for assessment of cholangiocarcinoma.

**FIGURE 6:**
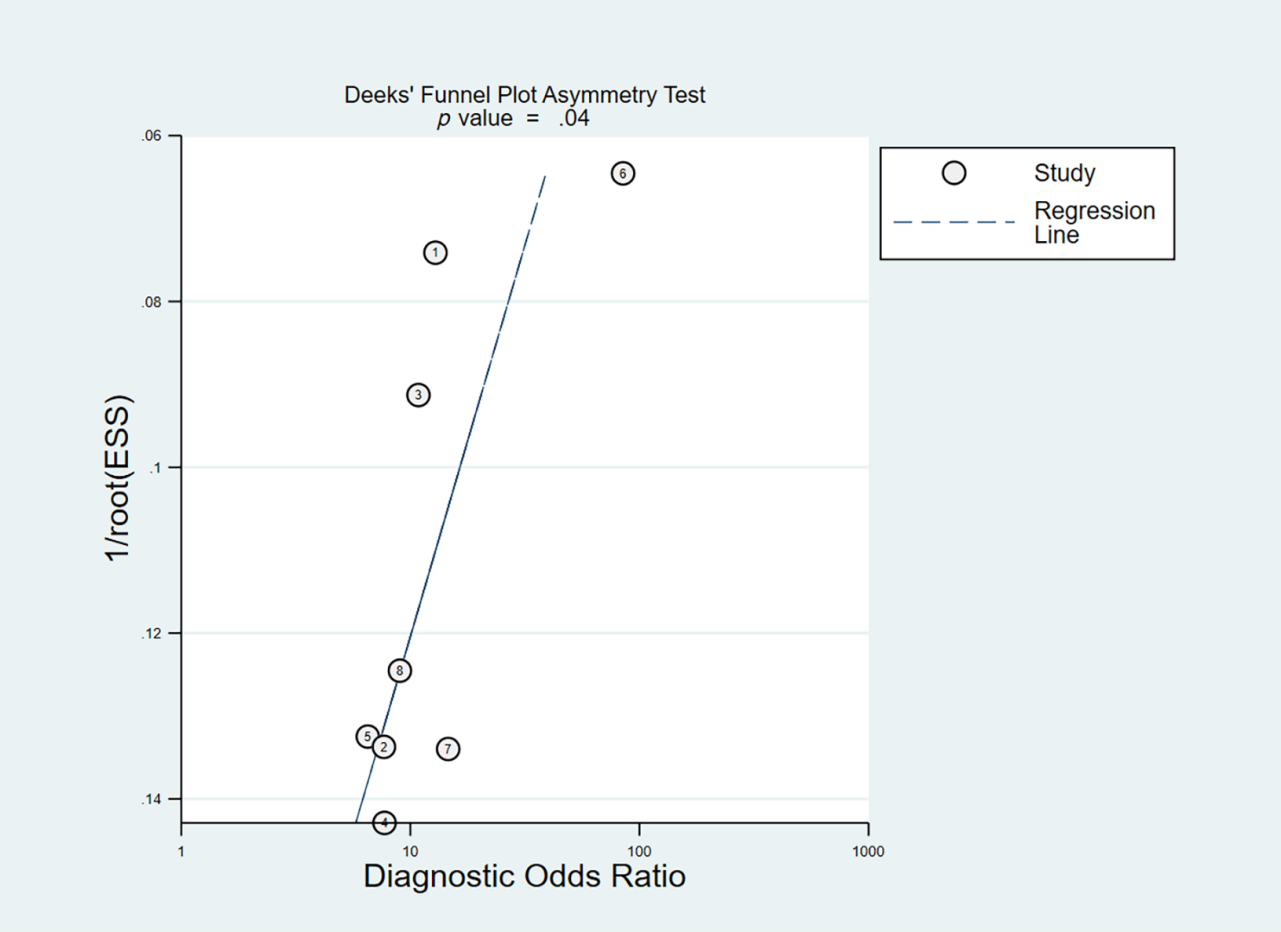
Deeks' funnel plot asymmetry test.

Based on individual study reports, the lncRNA PCAT1 had the highest sensitivity in diagnosing CCA,^35^ with a sensitivity of 87%; however, this lncRNA had a specificity of 69%. Regarding the highest specificity, the combination of PCAT1, MALAT1, and CPS1IT1 had an overall specificity of 93%.^33^ This combination exhibited a high sensitivity of 85% as well. Pooled sensitivity and specificity of prognostic or diagnostic accuracy of measurement of lncRNAs for assessment of CCA were 0.80 (95% CI: 0.75–0.84; *I* ^2^ = 8.39, *p* = .37) and 0.77 (95% CI: 0.68–0.84; *I* ^2^ = 73.61%, *p* < .01), respectively (Figure 3). Furthermore, its pooled positive and negative likelihood ratios were found to be 3.42 (95% CI: 2.35–4.98) and 0.26 (95% CI: 0.20–0.35), respectively (Figure 4). The AUC of the SROC was 0.83 (95% CI: 0.80–0.86) (Figure 5). Finally, the evaluation of publication bias based on the Deeks' funnel plot asymmetry test was performed, and it demonstrated the likeliness of publication bias in this meta‐analysis (*p* = .04) (Figure 6).

#### 3.3.2 Overall survival

**FIGURE 7:**
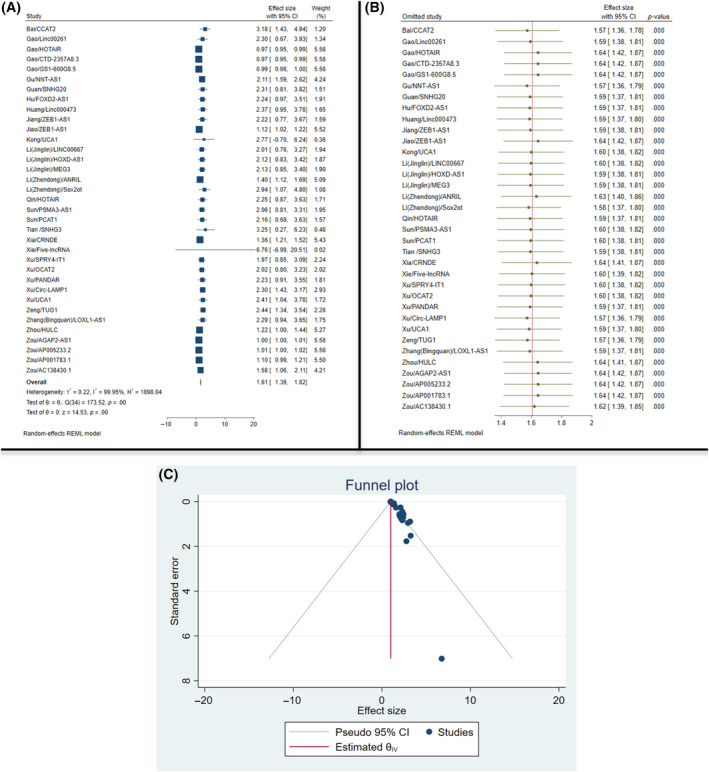
Subgroup analysis of the overall survival of the patients; (A): forest plot; (B): leave‐one‐out chart; (C): funnel plot.

Overall analysis indicated that measurement of lncRNAs for assessment of CCA significantly improves OS (effect size 1.61, 95% CI: 1.39–1.82; *I* ^2^ = 99.99, *p* < .01) (Figure 7A). CCAT2 was the lncRNA with the highest significant effect on overall survival (effect size 3.18, 95% CI: 1.43–4.94).^14^ The leave‐one‐out analysis of the OS is presented in Figure 7B. As demonstrated, the removal of none of the studies could affect the overall result significantly. The visual inspection of the OS funnel plot regarding the publication bias is presented in Figure 7C. There was no asymmetry in the funnel plot, presenting no chance of significant publication bias.

#### 3.3.3 Progression‐free survival

**FIGURE 8:**
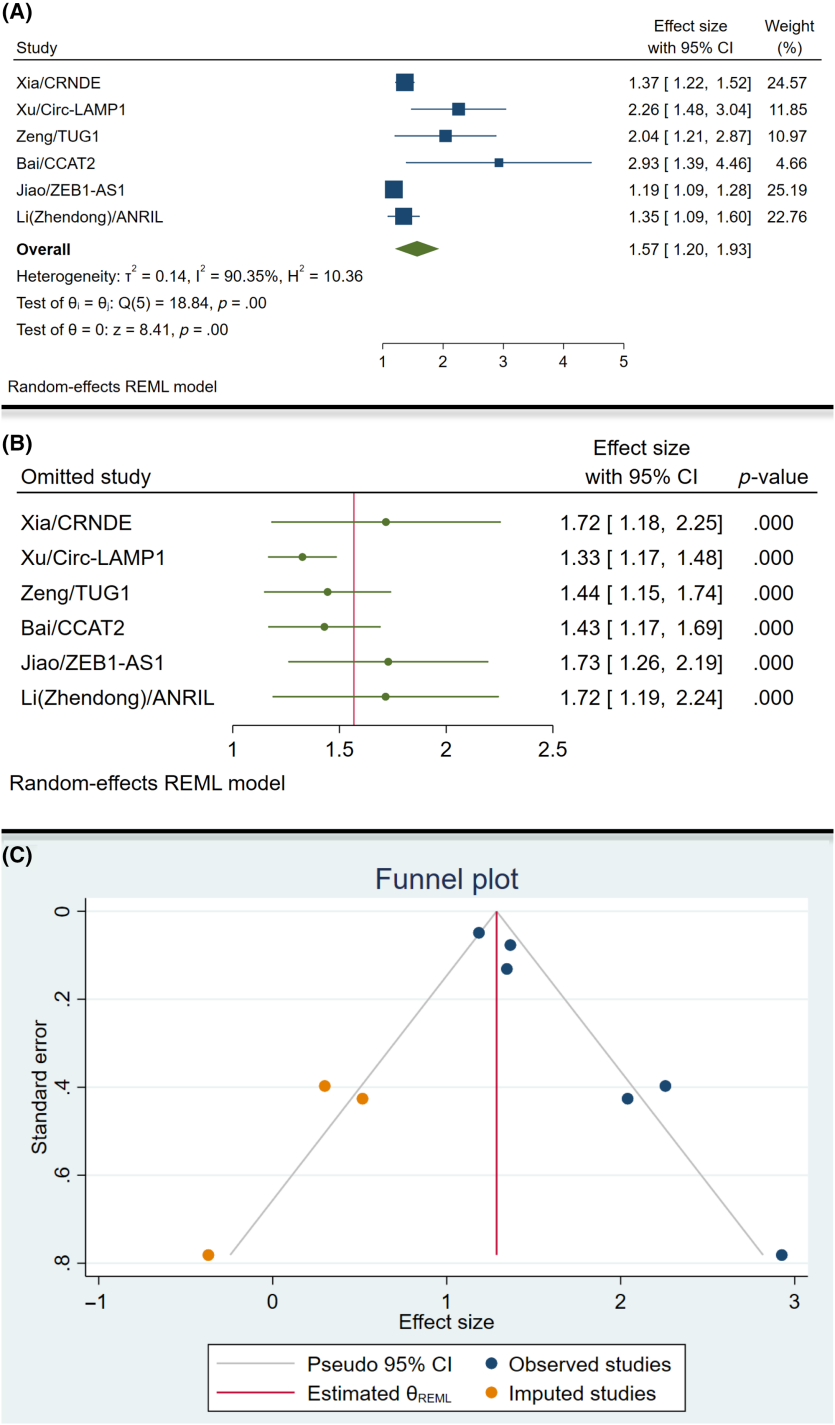
Subgroup analysis of the progression‐free survival of the patients; (A): forest plot; (B): leave‐one‐out chart; (C): funnel plot.

Based on Bai et al. study,^14^ CCAT2 lncRNA was associated with the highest overall effect size on PFS (effect size 2.93, 95% CI: 1.39–4.46). The overall analysis showed that the PFS was significantly improved by the measurement of lncRNAs for assessment of CCA (effect size 1.57, 95% CI: 1.20–1.93; *I* ^2^ = 90.35, *p* = .00) (Figure 8A). The leave‐one‐out analysis of the OS is shown in Figure 8B, which shows no effect on overall effect size by removal of each study. The visual inspection of the PFS funnel plot regarding the publication bias is shown in Figure 8C. There was an asymmetry in the funnel plot as three imputed studies are shown. Adding these three studies resulted in an overall insignificant result (effect size 1.29, 95% CI: 0.78–1.79).

## 4 DISCUSSION

This systematic review/meta‐analysis yielded 33 studies comprised of 2677 participants. The analyzed data revealed that measuring the levels of different types of lncRNAs plays a paramount role in diagnosing CCA. Also, it has a valuable prognostic value in the course of CCA in patients. The pooled AUC was 0.79, with a sensitivity of 0.80 and specificity of 0.77. LncRNA expression was also associated with improved overall and progression‐free survival in CCA patients. Among the lncRNAs, the combination of PCAT1, metastasis‐associated lung adenocarcinoma transcript 1 (MALAT1), and CPS1IT1 had the highest AUC for the diagnosis of CCA, and CCAT2 had the highest effect on overall survival and PFS. Hence, these are ideal biomarkers for further investigation in future studies.

Due to diagnosis at advanced stages,^4^ the 5‐year survival rate of those diagnosed with CCA is reported to be less than 30%,^47^ and the median overall survival of CCA is reported to be 11.7 months, which is far from satisfactory.^1^, ^48^ Therefore, an early diagnosis of CCA can improve its prognosis greatly, which can be achieved by measuring proper biomarkers. LncRNAs are related to various pathophysiological processes by regulation of gene expression through different mechanisms.^49^

TUG1, HOTAIR, PANDAR, and ZFAS1 are some of the most important types of lncRNAs, which play vital roles in multiple types of cancerous tumors.^40^, ^50^, ^51^, ^52^ In the investigation by Li et al.^28^ the Sox2 overlapping transcript (Sox2ot) was evaluated, which was overexpressed in CCA. As a result, Sox2ot had an association with the prognosis of CCA patients. The lncRNA XIST was among the top differentially expressed LncRNAs in the heatmap analysis. Aligning with our meta‐analysis, this indicates that upregulated XIST has potential utility as a diagnostic biomarker in CCA.

Among the lncRNAs investigated in this study, MALAT1, also known as nuclear‐enriched transcript 2 (NEAT2) showed high diagnostic value for CCA. Originally, MALAT1 was discovered in non‐small cell lung cancer and was determined as a prognostic marker for metastasis in these patients.^53^ However, its association with several other human cancers has been shown.^54^, ^55^, ^56^ This lncRNA affects several processes in cells, including proliferation, apoptosis, cell‐cycle progression, and cell growth.^57^ In our study, the combination of PCAT1, MALAT1, and CPS1IT1 showed the highest diagnostic ability among all lncRNAs. In this regard, Tan et al. showed that the MALAT1/miR‐204/CXCR4 axis plays an important role in human hilar CCA by affecting cell growth, invasion, and migration, hence, could have a potential therapeutic application.^58^

LncRNA CCAT1 (colon cancer‐associated transcript1) is another type of lncRNA that was shown to have upregulated expression in colon cancer.^59^ Moreover, it has been found that CCAT1 is being upregulated in several cancers and is related to tumor progression stages, such as differentiation, proliferation, and chemoresistance.^60^, ^61^, ^62^ Jiang et al.^63^ investigated the CCAT1 expression association with CCA, and found significantly higher levels of CCAT1 in tumor tissues, compared to normal tissues. This showed that upregulated expression of CCAT1 was associated with CCA in terms of lymph node invasion, advanced TNM stage, and also poor histological differentiation. They concluded that lncRNA CCAT1 is eligible to be considered as a biomarker in CCA. Similarly, Bai et al. found promising results for CCAT2 in use as a prognostic marker in CCA.^14^

LncRNA carbamoyl‐phosphate synthetase 1 intronic transcript 1 (CPS1IT1) was another highly ranked lncRNA for CCA, based on our findings. This lncRNA maps to chromosome 17q24.3 within an intron of the gene encoding CPS1^64^ and was shown to be downregulated in several cancers and act as a tumor suppressor in these cancers.^65^, ^66^, ^67^, ^68^ In addition to the diagnostic studies included in our systematic review, CPS1IT1 was upregulated in intrahepatic CCA.^69^ There is a need for further studies regarding its use in CCA.

Despite systematically reviewing the role of lncRNA in CCA, our study has several limitations. First, significant heterogeneity was observed across studies. In addition, all included studies were from China, hence, these results cannot be extrapolated into other regions and the possible effects of ethnicity and region are not yet shown. Investigation of these lncRNAs in other populations is highly warranted in order to better clarify these results. Third, most of the lncRNAs were not investigated very frequently and hence, future studies should address the best diagnostic lncRNAs specifically with the aim of use in clinical settings. Fourth, since we did not include case reports, some of the rare lncRNAs that could be potentially useful in diagnosis might be missed. Also, as most of the individual studies did not report clinicopathological features of CCA and there was high heterogeneity among the studies, we were not able to assess the association of these features and lncRNAs. So, our study is hypothesis‐making for the use of lncRNAs in CCA and further studies are needed to investigate these associations in detail. Finally, while our results suggest the effectiveness of measuring lncRNAs in CCA, top lncRNAs were only reported based on individual studies and there is a need for further investigation of these markers such as CCAT2.

## 5 CONCLUSION

In conclusion, our systematic review found that lncRNAs, especially when measured as multi‐lncRNA panels, show promise as diagnostic and prognostic biomarkers in CCA patients. However, future large‐scale studies are needed to validate the best lncRNA signatures for clinical use. These results can have clinical applicability due to the determination of the diagnostic ability of lncRNAs in CCA. It is of higher importance as there is no definitive diagnostic tool for early detection of CCA. In most cases, increased levels of lncRNAs were associated with larger tumor size, increased metastatic activity, and a worse prognosis. However, further high‐quality studies assessing larger sample sizes and also in different countries are suggested to be conducted in order to achieve a comprehensive conclusion regarding the utility of measuring lncRNAs as a powerful tool for the assessment of CCA.

## AUTHOR CONTRIBUTIONS

**Parnian Shobeiri:** Conceptualization (equal); formal analysis (equal); visualization (equal); writing – original draft (equal). **Razman Arabzadeh Bahri:** Conceptualization (equal); formal analysis (equal); visualization (equal); writing – original draft (equal). **Mohamad Mehdi Khadembashiri:** Data curation (equal); writing – original draft (equal). **Mohamad Amin Khadembashiri:** Data curation (equal); writing – original draft (equal). **Saba Maleki:** Data curation (equal); writing – original draft (equal). **Mohammad Eslami:** Writing – review and editing (equal). **Marzie Khalili Dehkordi:** Writing – review and editing (equal). **Amir Hossein Behnoush:** Writing – review and editing (equal). **Nima Rezaei:** Supervision (equal); writing – review and editing (equal).

## CONFLICT OF INTEREST STATEMENT

None.

## ETHICS STATEMENT

Not applicable.

## Supporting information


**Supplementary Table 1.** Search details.
**Supplementary Table 2.** Quality of included studies based on QUADAS‐2 and QUIPS criteria.

## Data Availability

Data sharing is not applicable to this article as no new data were created or analyzed in this study.
