# Supplementary material for: Role of long non‐coding RNAs in cholangiocarcinoma: A systematic review and meta‐analysis
Source: Cancer Rep (Hoboken). 2024 Mar 22;7(3):e2029. doi: 10.1002/cnr2.2029 (PMC10959185; doi:10.1002/cnr2.2029)
Supplement: Supplementary file 1 — Supplementary Table 1. Search details. Supplementary Table 2. Quality of included studies based on QUADAS‐2 and QUIPS criteria. [file CNR2-7-e2029-s001.docx]

**Supplementary Materials**

***Supplementary Table 1.*** Search details

| **Query** | | **Results**  **(Oct 20, 2022)** |
| --- | --- | --- |
| **PubMed** | | |
| #1 | (“bile duct carcinoma” OR “bile duct carcinoma” OR “bile tract carcinoma” OR “biliary carcinoma” OR “biliary duct carcinoma” OR “biliary tract carcinoma” OR “carcinoma, intrahepatic bile duct” OR “cholangiocarcinoma” OR “cholangiocellular carcinoma” OR “cholangiolar carcinoma” OR “extrahepatic bile duct carcinoma” OR “extrahepatic biliary duct carcinoma” OR “extrahepatic cholangiocarcinoma” OR “gall duct carcinoma” OR “hilar cholangiocarcinoma” OR “intrahepatic bile duct carcinoma” OR “intrahepatic biliary duct carcinoma” OR “intrahepatic cholangiocarcinoma” OR “malignant cholangioma” OR “perihilar bile duct carcinoma” OR “perihilar biliary duct carcinoma” OR “perihilar cholangiocarcinoma”) | 21,749 |
| #2 | (“long untranslated rna” OR “rna, long noncoding” OR “rna, long untranslated” OR “large intergenic non coding rna” OR “large intergenic non protein coding rna” OR “large intergenic noncoding rna” OR “linc rna” OR “lincrna” OR “lnc rna” OR “lncrna” OR “long ncrna” OR “long ncrnas” OR “long non coding rna” OR “long non protein coding rna” OR “long noncoding rna” OR “long untranslated rna”) | 42,283 |
| #3 | #1 AND #2 | ***161*** |
| **Embase** | | |
| #1 | ('bile duct carcinoma'/exp OR 'bile duct carcinoma' OR 'bile tract carcinoma' OR 'biliary carcinoma' OR 'biliary duct carcinoma' OR 'biliary tract carcinoma' OR 'carcinoma, intrahepatic bile duct' OR 'cholangiocarcinoma' OR 'cholangiocellular carcinoma' OR 'cholangiolar carcinoma' OR 'extrahepatic bile duct carcinoma' OR 'extrahepatic biliary duct carcinoma' OR 'extrahepatic cholangiocarcinoma' OR 'gall duct carcinoma' OR 'hilar cholangiocarcinoma' OR 'intrahepatic bile duct carcinoma' OR 'intrahepatic biliary duct carcinoma' OR 'intrahepatic cholangiocarcinoma' OR 'malignant cholangioma' OR 'perihilar bile duct carcinoma' OR 'perihilar biliary duct carcinoma' OR 'perihilar cholangiocarcinoma') | 39,931 |
| #2 | ('long untranslated rna'/exp OR 'rna, long noncoding' OR 'rna, long untranslated' OR 'large intergenic non coding rna' OR 'large intergenic non protein coding rna' OR 'large intergenic noncoding rna' OR 'linc rna' OR 'lincrna' OR 'lnc rna' OR 'lncrna' OR 'long ncrna' OR 'long ncrnas' OR 'long non coding rna' OR 'long non protein coding rna' OR 'long noncoding rna' OR 'long untranslated rna') | 51,333 |
| #3 | #1 AND #2 | ***301*** |
| **TOTAL** | | ***462*** |
| **TOTAL AFTER REMOVING DUPLICATES** | | ***316*** |

***Supplementary Table 2.*** *Quality of included studies based on QUADAS-2 and QUIPS criteria*

| **Study (Author, year)** | **1. Study Participation** | **2. Study Attrition** | **3. Prognostic Factor Measurement** | **4. Outcome Measurement** | **5. Study Confounding** | **6. Statistical Analysis and Reporting** |
| --- | --- | --- | --- | --- | --- | --- |
| Canghai Guan, 2020 (16) | low | low | low | low | low | low |
| Bo-Wei Han, 2018 (17) | low | low | low | low | low | low |
| Lining Huang, 2020 (19) | low | low | low | low | low | low |
| Yulei Gu, 2019 (15) | unclear | low | low | low | low | low |
| Juan-Guo Bai, 2018 (11) | low | low | low | low | low | low |
| Meihong Gao, 2022 (13) | high | unclear | unclear | low | unclear | low |
| Jianjun Gao, 2020 (12) | low | low | low | low | low | low |
| Zengtao Hu, 2022 (18) | low | low | low | low | low | low |
| Xianxiu Ge, 2017 (14) | low | low | unclear | low | low | low |
| Jian Shi, 2018 (30) | low | high | low | low | unclear | low |
| Lei Kong, 2019 (21) | low | low | low | low | unclear | low |
| Zhendong Li, 2018 (26) | low | low | low | low | low | low |
| Zhenglong Li, 2018 (25) | low | low | low | low | low | low |
| Jinglin Li, 2020 (24) | low | low | low | low | low | low |
| Chunying Luo, 2021 (28) | high | low | low | low | low | low |
| Jinglin Li, 2019 (23) | low | low | low | low | low | low |
| Jinglin Li, 2021 (22) | low | low | low | low | low | low |
| Zhanqiang Liang, 2019 (27) | low | low | low | low | unclear | low |
| Dongsheng Sun, 2021 (32) | unclear | low | low | low | low | low |
| Dongsheng Sun, 2022 (31) | low | low | low | low | low | low |
| Xingming Jiang, 2020 (20) | low | high | low | low | unclear | low |
| Wei Qin, 2018 (29) | low | unclear | low | low | low | low |
| Daguang Tian, 2019 (33) | high | low | low | low | high | low |
| Xiu‑Liang Xia, 2018 (34) | low | unclear | high | low | high | low |
| Yi, Xu, 2017 (37) | low | low | low | low | low | low |
| Yi, Xu, 2018 (38) | low | low | unclear | low | low | low |
| Yi, Xu, 2021 (36) | low | low | low | unclear | unclear | low |
| Yi, Xu, 2017 (39) | low | low | low | low | low | low |
| Yi, Xu, 2018 (40) | low | low | low | low | low | low |
| Bing Zeng, 2017 (41) |  |  |  |  |  |  |
| Dongkai Zhou, 2019 (43) | low | low | low | low | low | low |
| Xiaozai Xie, 2021 (35) | low | low | low | low | low | low |
| Bingquan Zhang, 2019 (42) | low | low | low | low | low | low |
